# Supplementary material for: Modeling glioblastoma heterogeneity as a dynamic network of cell states
Source: Mol Syst Biol. 2021 Sep 16;17(9):e10105. doi: 10.15252/msb.202010105 (PMC8444284; doi:10.15252/msb.202010105)
Supplement: Supplementary file 6 — Source Data for Figure 5 [file MSB-17-e10105-s004.zip › Figure5A_sourcedata/GSEA_3017/nosigdb_stateC.GseaPreranked.1622795002785/RICHARDS_NOWAKOWSKI_SCIENCE_2017_ASTROCYTE_UPREG.html]

Details for gene set RICHARDS\_NOWAKOWSKI\_SCIENCE\_2017\_ASTROCYTE\_UPREG[GSEA]

|  || Dataset | state13017 |
| Phenotype | NoPhenotypeAvailable |
| Upregulated in class | na\_neg |
| GeneSet | RICHARDS\_NOWAKOWSKI\_SCIENCE\_2017\_ASTROCYTE\_UPREG |
| Enrichment Score (ES) | -0.49444634 |
| Normalized Enrichment Score (NES) | -3.4200554 |
| Nominal p-value | 0.0 |
| FDR q-value | 0.0 |
| FWER p-Value | 0.0 |
Table: GSEA Results Summary

  

Fig 1: Enrichment plot: RICHARDS\_NOWAKOWSKI\_SCIENCE\_2017\_ASTROCYTE\_UPREG      
 Profile of the Running ES Score & Positions of GeneSet Members on the Rank Ordered List

  

| PROBE | GENE SYMBOL | GENE\_TITLE | RANK IN GENE LIST | RANK METRIC SCORE | RUNNING ES | CORE ENRICHMENT || 1 | MAGI1 |  |  | 6 | 0.652 | 0.0025 | No |
| 2 | ZBTB20 |  |  | 30 | 0.482 | -0.0333 | No |
| 3 | CDCA7L |  |  | 37 | 0.453 | -0.0353 | No |
| 4 | ETV1 |  |  | 44 | 0.438 | -0.0376 | No |
| 5 | GAS1 |  |  | 64 | 0.384 | -0.0675 | No |
| 6 | RHOBTB3 |  |  | 124 | 0.313 | -0.1803 | No |
| 7 | SCN1A |  |  | 195 | -0.250 | -0.3169 | No |
| 8 | ARHGAP12 |  |  | 207 | -0.254 | -0.3335 | No |
| 9 | HEPACAM |  |  | 216 | -0.257 | -0.3440 | No |
| 10 | FERMT2 |  |  | 227 | -0.259 | -0.3584 | No |
| 11 | SAT1 |  |  | 241 | -0.262 | -0.3789 | No |
| 12 | GNG7 |  |  | 253 | -0.264 | -0.3953 | No |
| 13 | NPAS3 |  |  | 256 | -0.265 | -0.3934 | No |
| 14 | ID4 |  |  | 261 | -0.267 | -0.3955 | No |
| 15 | PPIB |  |  | 265 | -0.268 | -0.3955 | No |
| 16 | GATM |  |  | 272 | -0.270 | -0.4016 | No |
| 17 | HSD17B12 |  |  | 277 | -0.271 | -0.4036 | No |
| 18 | HEATR5A |  |  | 278 | -0.271 | -0.3975 | No |
| 19 | DTNA |  |  | 281 | -0.271 | -0.3954 | No |
| 20 | TGFB2 |  |  | 285 | -0.272 | -0.3954 | No |
| 21 | ABHD2 |  |  | 296 | -0.275 | -0.4095 | No |
| 22 | SLC1A3 |  |  | 307 | -0.281 | -0.4235 | No |
| 23 | LIFR |  |  | 311 | -0.282 | -0.4232 | No |
| 24 | PLEKHH2 |  |  | 319 | -0.284 | -0.4310 | No |
| 25 | SPATS2L |  |  | 339 | -0.290 | -0.4631 | No |
| 26 | DPP6 |  |  | 345 | -0.296 | -0.4666 | No |
| 27 | DLC1 |  |  | 347 | -0.296 | -0.4619 | No |
| 28 | ABLIM1 |  |  | 348 | -0.297 | -0.4552 | No |
| 29 | MEST |  |  | 352 | -0.299 | -0.4545 | No |
| 30 | LRP1 |  |  | 366 | -0.304 | -0.4741 | No |
| 31 | KANK1 |  |  | 374 | -0.308 | -0.4813 | No |
| 32 | FOS |  |  | 377 | -0.309 | -0.4784 | No |
| 33 | CSPG5 |  |  | 381 | -0.310 | -0.4775 | No |
| 34 | SLC2A1 |  |  | 383 | -0.311 | -0.4725 | No |
| 35 | IGFBP5 |  |  | 384 | -0.311 | -0.4655 | No |
| 36 | DPF3 |  |  | 390 | -0.316 | -0.4685 | No |
| 37 | LRRC4C |  |  | 394 | -0.318 | -0.4674 | No |
| 38 | S100A13 |  |  | 396 | -0.319 | -0.4622 | No |
| 39 | TIMP1 |  |  | 399 | -0.320 | -0.4590 | No |
| 40 | ZFP36L2 |  |  | 401 | -0.321 | -0.4538 | No |
| 41 | FGFR3 |  |  | 403 | -0.322 | -0.4486 | No |
| 42 | SLC4A4 |  |  | 415 | -0.332 | -0.4634 | No |
| 43 | PMP2 |  |  | 424 | -0.337 | -0.4721 | No |
| 44 | VEGFA |  |  | 436 | -0.345 | -0.4867 | Yes |
| 45 | PCDH9 |  |  | 439 | -0.345 | -0.4829 | Yes |
| 46 | FAT1 |  |  | 441 | -0.346 | -0.4771 | Yes |
| 47 | RAB31 |  |  | 445 | -0.349 | -0.4753 | Yes |
| 48 | BCAN |  |  | 449 | -0.351 | -0.4735 | Yes |
| 49 | LUZP2 |  |  | 450 | -0.352 | -0.4655 | Yes |
| 50 | IDI1 |  |  | 451 | -0.353 | -0.4576 | Yes |
| 51 | SERPINE2 |  |  | 452 | -0.355 | -0.4495 | Yes |
| 52 | NEK6 |  |  | 453 | -0.355 | -0.4415 | Yes |
| 53 | RHOC |  |  | 455 | -0.359 | -0.4354 | Yes |
| 54 | SLITRK2 |  |  | 458 | -0.361 | -0.4313 | Yes |
| 55 | EDNRB |  |  | 462 | -0.367 | -0.4291 | Yes |
| 56 | NTRK3 |  |  | 464 | -0.368 | -0.4229 | Yes |
| 57 | ALCAM |  |  | 467 | -0.370 | -0.4186 | Yes |
| 58 | B2M |  |  | 468 | -0.371 | -0.4102 | Yes |
| 59 | HES1 |  |  | 470 | -0.373 | -0.4038 | Yes |
| 60 | RAB13 |  |  | 482 | -0.388 | -0.4174 | Yes |
| 61 | OLIG1 |  |  | 487 | -0.393 | -0.4166 | Yes |
| 62 | C1orf61 |  |  | 491 | -0.396 | -0.4137 | Yes |
| 63 | FABP7 |  |  | 495 | -0.411 | -0.4106 | Yes |
| 64 | AGT |  |  | 497 | -0.412 | -0.4033 | Yes |
| 65 | OLIG2 |  |  | 499 | -0.415 | -0.3959 | Yes |
| 66 | FJX1 |  |  | 501 | -0.418 | -0.3885 | Yes |
| 67 | TGIF1 |  |  | 502 | -0.421 | -0.3790 | Yes |
| 68 | JUN |  |  | 503 | -0.425 | -0.3694 | Yes |
| 69 | SCD |  |  | 504 | -0.431 | -0.3596 | Yes |
| 70 | F3 |  |  | 505 | -0.432 | -0.3498 | Yes |
| 71 | SLC25A18 |  |  | 507 | -0.433 | -0.3421 | Yes |
| 72 | SCRG1 |  |  | 508 | -0.434 | -0.3323 | Yes |
| 73 | DBI |  |  | 514 | -0.440 | -0.3325 | Yes |
| 74 | CD9 |  |  | 515 | -0.443 | -0.3225 | Yes |
| 75 | PTN |  |  | 517 | -0.446 | -0.3144 | Yes |
| 76 | CA2 |  |  | 518 | -0.448 | -0.3043 | Yes |
| 77 | CST3 |  |  | 520 | -0.452 | -0.2961 | Yes |
| 78 | LYPD1 |  |  | 521 | -0.454 | -0.2858 | Yes |
| 79 | MSMO1 |  |  | 525 | -0.460 | -0.2815 | Yes |
| 80 | HMGCS1 |  |  | 529 | -0.466 | -0.2771 | Yes |
| 81 | COL6A1 |  |  | 534 | -0.479 | -0.2744 | Yes |
| 82 | ITGA6 |  |  | 535 | -0.479 | -0.2636 | Yes |
| 83 | TTYH1 |  |  | 540 | -0.496 | -0.2605 | Yes |
| 84 | SEMA6D |  |  | 541 | -0.500 | -0.2492 | Yes |
| 85 | LRRN3 |  |  | 543 | -0.504 | -0.2398 | Yes |
| 86 | HOPX |  |  | 546 | -0.513 | -0.2323 | Yes |
| 87 | GJA1 |  |  | 552 | -0.527 | -0.2305 | Yes |
| 88 | SPARCL1 |  |  | 557 | -0.544 | -0.2263 | Yes |
| 89 | S100B |  |  | 561 | -0.561 | -0.2198 | Yes |
| 90 | CREM |  |  | 562 | -0.563 | -0.2070 | Yes |
| 91 | TNC |  |  | 564 | -0.572 | -0.1961 | Yes |
| 92 | PCDH10 |  |  | 565 | -0.573 | -0.1832 | Yes |
| 93 | MT2A |  |  | 566 | -0.575 | -0.1702 | Yes |
| 94 | S100A16 |  |  | 569 | -0.582 | -0.1611 | Yes |
| 95 | ITPR2 |  |  | 572 | -0.634 | -0.1508 | Yes |
| 96 | FGFBP3 |  |  | 573 | -0.644 | -0.1362 | Yes |
| 97 | GFAP |  |  | 574 | -0.659 | -0.1213 | Yes |
| 98 | VIM |  |  | 578 | -0.674 | -0.1122 | Yes |
| 99 | NEAT1 |  |  | 579 | -0.683 | -0.0967 | Yes |
| 100 | CLU |  |  | 581 | -0.690 | -0.0832 | Yes |
| 101 | CYR61 |  |  | 584 | -0.714 | -0.0711 | Yes |
| 102 | ATP1B2 |  |  | 585 | -0.714 | -0.0550 | Yes |
| 103 | PMP22 |  |  | 590 | -0.837 | -0.0442 | Yes |
| 104 | MT3 |  |  | 591 | -0.837 | -0.0253 | Yes |
| 105 | FABP5 |  |  | 596 | -1.477 | 0.0000 | Yes |
Table: GSEA details [plain text format]

  

Fig 2: RICHARDS\_NOWAKOWSKI\_SCIENCE\_2017\_ASTROCYTE\_UPREG: Random ES distribution      
 Gene set null distribution of ES for **RICHARDS\_NOWAKOWSKI\_SCIENCE\_2017\_ASTROCYTE\_UPREG**
